# A Cross-Sectional Study Revealed a Low Prevalence of SARS-CoV-2 Infection among Asymptomatic University Students in Tripoli, North Lebanon

**DOI:** 10.3390/pathogens13100872

**Published:** 2024-10-03

**Authors:** Rayane Rafei, Layla Tajer, Dalal Nour, Karen Abboud, Dima Ankoud, Marwan Osman, Marielle Bedotto, Mohamad Bachar Ismail, Fouad Dabboussi, Philippe Colson, Monzer Hamze

**Affiliations:** 1 Laboratoire Microbiologie Santé et Environnement (LMSE), Doctoral School for Science & Technology, Faculty of Public Health, Lebanese University, Tripoli 1300, Lebanon; 2 Microbes Evolution Phylogeny and Infections (MEPHI), Institut de Recherche pour le Développement (IRD), Aix-Marseille University, 27 Boulevard Jean Moulin, 13005 Marseille, France; 3 Department of Neurosurgery, Yale School of Medicine, Yale University, New Haven, CT 06510, USA; 4 Assistance Publique-Hôpitaux de Marseille (AP-HM), 264 Rue Saint-Pierre, 13005 Marseille, France; 5 Faculty of Sciences, Lebanese University, Tripoli 1300, Lebanon; 6 IHU Méditerranée Infection, 19-21 Boulevard Jean Moulin, 13005 Marseille, France

**Keywords:** SARS-CoV-2, COVID-19, asymptomatic carriers, Omicron BA.2, Lebanon, prevalence

## Abstract

This study aimed to investigate the SARS-CoV-2 infection prevalence among >18-year-old students in the Faculty of Public Health and Faculty of Sciences at the Lebanese University in Tripoli, Northern Lebanon, in June 2023 and to characterize the circulating Omicron subvariants. Out of 357 participants, only 2 (0.56%) tested positive by qPCR, corresponding to 0.61% (2/326) of asymptomatic students. One case tested positive with a qPCR targeting the Omicron BA.2 variant. These findings indicate a low incidence at that time and emphasize the interest of SARS-CoV-2 surveillance among students.

## 1. Introduction

Since its emergence from Wuhan, China, in December 2019, SARS-CoV-2 (Severe Acute Respiratory Syndrome Coronavirus 2), the causative agent of COVID-19 (Coronavirus-2019) disease, has continued to spread globally [1]. As of 8 November 2023, the World Health Organization (WHO) reported a cumulative total of 771,820,937 confirmed cases of COVID-19 and 6,978,175 deaths [2]. To address the SARS-CoV-2 pandemic, the Lebanese government implemented several stringent public health and social measures aimed at controlling the virus’s transmission. These measures included closing certain entry points, limiting travel, enforcing national lockdowns, mandating face mask usage, instituting social distancing protocols, and requiring quarantine following potential exposure to confirmed or suspected COVID-19 cases [3]. Additionally, alongside these control efforts, the Ministry of Public Health launched a vaccination campaign, administering a total of 5,814,699 doses as of December 2023 [2,4]. Adhering to these safety guidelines, the country controlled the frequency of hospitalizations and the rapid increase in cases [3].

Numerous studies conducted worldwide have investigated the impact and prevalence of COVID-19 in crowded places such as universities [5], which could be considered hotspots for COVID-19 transmission. However, to our knowledge, this topic has not been explored yet in Lebanon. Research in Lebanon has primarily focused on public awareness of COVID-19 symptoms, preventive measures, and vaccination. Studies have also examined the levels of stress, anxiety, and depression, along with influential risk factors affecting the adoption of COVID-19 preventive practices [6,7,8,9,10,11,12,13,14,15]. Additionally, several studies have been conducted to investigate the variants in Lebanon [16,17,18]. For instance, a study conducted by Nour et al. described the epidemiological dynamics of SARS-CoV-2 during the first two years of the pandemic in Lebanon, which played a pivotal role in the spread of the B.1.398 variant to various countries, facilitating its dissemination across continents [18]. Additionally, other studies assessed the SARS-CoV-2 antibodies and their levels in the Lebanese population [19,20].

In Lebanon, although surveillance of SARS-CoV-2 was far from being on a tight rein before April 2023, it eroded thereafter. No systematic surveillance was implemented on symptomatic patients and was associated with a notable gap in understanding the prevalence of SARS-CoV-2 infection among asymptomatic carriers, especially in crowded settings. This study aims to determine the prevalence of SARS-CoV-2 among university students and to describe the circulating SARS-CoV-2 Omicron subvariants, especially during June when there was a dearth of prevalence data for Tripoli, Northern Lebanon, or Lebanon. Such surveys are also needed to assess the adequacy of in-person teaching adopted after more than two years of online instruction.

## 2. Materials and Methods

This cross-sectional study was validated by the ethics committee of the Azm Center for Research in Biotechnology and Its Applications (CE-EDST-2-2023), Lebanese University. It was conducted between 19 and 21 June 2023 in two faculties (Faculty of Public Health and Faculty of Sciences, Branch III, comprising 510 and 3700 students, respectively) of the Lebanese University in Tripoli, Northern Lebanon. A sample size of 134 was calculated using the Raosoft Inc., 2004 (http://www.raosoft.com/samplesize.html (accessed on 10 June 2023)), taking into account an average PCR positivity rate of 9.92% for the first four months of 2023. These data were obtained by the Ministry of Public Health in Lebanon, as the daily monitoring of cases was conducted throughout these months (http://www.moph.gov.lb (accessed on 2 January 2024)). The students in the two faculties were recruited in person after the researchers explained the study’s aims. Nasopharyngeal swabs were collected anonymously and randomly from students after obtaining their informed consent. Participation was voluntary, and students were fully informed of their right to withdraw from the study at any time without consequence. RNA was extracted from these samples using the Viral DNA/RNA Kit (CWBIO Biosciences, Taizhou, China) according to the manufacturer’s protocol. SARS-CoV-2 infection was detected using the TaqPath COVID-19 CE-IVD RT-PCR kit (Thermo Fisher Scientific, Waltham, MA, USA). For positive samples, four additional real-time reverse transcription PCR (qPCR) tests using in-house primers and probes (Table S1) were performed to identify the specific SARS-CoV-2 Omicron subvariant using primers targeting the BA.1, BA.2, BA.4, and BA.5 subvariants. Sociodemographic and health-related data for the students were collected using a questionnaire in Arabic. The sociodemographic section includes variables such as age, sex, marital status, education level, residential location, family type, and occupation. The health-related section includes questions about symptomatic status, previous COVID-19 infection, chronic diseases, vaccination status, type of vaccine received, and treatment during COVID-19.

## 3. Results

A total of 357 participants were enrolled in this study, of whom 54.6% (*n* = 195) were from the Faculty of Sciences and 45.4% (*n* = 162) were from the Faculty of Public Health. They represented 5.3% and 31.8% of students from these faculties, respectively. The sample was also diverse, encompassing students enrolled in different disciplines and academic levels, which thus increases its representativeness of the student population. The COVID-19 vaccination rate was 58.3% among all enrolled students, and 40.1% of them were infected with SARS-CoV-2 at least once before this survey. A total of 31 (8.7%) students were presenting with respiratory symptoms, whereas 326 (91.3%) were asymptomatic (Table 1). The asymptomatic or symptomatic status of each individual was assigned based on answers given to the question “Are you currently experiencing respiratory infections?” in the questionnaire that was provided to each volunteer. However, only two (0.56%) samples tested positive for SARS-CoV-2, with cycle threshold (Ct) values from one patient for the ORF1ab, S, and N genes being 25, 25.59, and 23.3, respectively. Meanwhile, the Ct values for the ORF1ab, S, and N genes in another patient were 26.3, 26.53, and 25.8, respectively. Both positive samples were from the asymptomatic students’ group, in which the overall SARS-CoV-2 prevalence was 0.61% (2/326).

One of the two students tested positive for the Omicron BA.2 subvariant with this lineage’s specific qPCR, while the other yielded negative results in the four qPCR-specific tests for the Omicron subvariants. Both students fell within the 18–23 age group. The first case (qPCR Ct = 23.3 for the N gene), BA.2-infected, was a vaccinated student from the Faculty of Public Health, trained at three hospitals, and was exposed to a SARS-CoV-2-positive technical worker within 14 days before sampling. The other case (qPCR Ct = 25.8 for the N gene) was unvaccinated, from the Faculty of Sciences, and had not undergone medical training. Neither of the students traveled within the last 14 days nor reported SARS-CoV-2-positive cases in their families, but both frequented crowded places like the University and buses. Due to logistical reasons, the results were not communicated to the participants.

## 4. Discussion

This study was conducted during the first year of in-person teaching (2022–2023) at the Lebanese University, following two and a half years of online teaching. During this period, classrooms were crowded, and preventive measures and social distancing were minimal. Studies evaluating SARS-CoV-2 prevalence in such crowded settings, which are potential hotspots for transmission, are valuable.

Additionally, the Lebanese University is the only public university in Lebanon, and the two studied faculties located in Tripoli, Northern Lebanon, contain students from Lebanon, particularly from North Lebanon, representing thus the North Lebanese population. Therefore, the obtained results did not reflect only the situation in a specific crowded university but also in Tripoli and North Lebanon. It is also important to note that respiratory samples were collected in June 2023, a period when the average PCR positivity rate of SARS-CoV-2 was relatively low (3.4%) compared to other previous months (an average PCR positivity rate of 9.92% for the first four months of 2023). This may reflect the limited incidence of positive COVID-19 cases at that time or a lack of strong case tracking. According to WHO, there were 800 positive confirmed cases in May and 996 cases notified over just three tracking days in June. Confirmed cases in June were recorded as follows: 808 on June 11, 47 on June 18, and 141 on June 25. In July, however, no positive cases were reported [21].

Here, only two students (0.56%) tested positive for SARS-CoV-2, highlighting a very low infection rate. This result aligns with that of a study conducted at a Thai university during a similar period of the year, for which a prevalence of 1.2% was recorded [5]. This low infection rate indicates that SARS-CoV-2 circulated at a very low rate among tested students at the time of the present study, which is in agreement with the potential low incidence of SARS-CoV-2 in Lebanon in that period. This may not be explained by the summer season [22] as the epidemics with some previous SARS-CoV-2 VOC emerged during hot months (for instance, the Delta variant in the Northern hemisphere) and/or African countries (for instance, the Omicron BA.1 and BA.2 lineages). Additionally, the combined rate of previous vaccination and/or infection was 72.3% here, but immune responses toward SARS-CoV-2 were reported to be often overcome and to rapidly wan [23,24,25]. Of note, hesitancy was important among Lebanese adults toward receiving the COVID-19 vaccine [6]. Furthermore, the participation rate (8.47%) was very low, which could be attributed to the invasive nature of the sampling process and to concerns of stigma associated with being tested SARS-CoV-2-positive, particularly among symptomatic students, possibly explaining in part their low number (31 of all 357 students tested). In this view, the only two cases detected here were asymptomatic, but in a framework where most tested students were asymptomatic. Regardless, testing asymptomatic students was useful here and was reported to be valuable, as previously [26] and as asymptomatic infections are frequent [27]. Additionally, the population of the study was younger adults, who generally have stronger immune responses against infections and are less affected by comorbidities, resulting in milder or asymptomatic infections [28,29,30]. Age is also associated with a decreased vaccine-induced immune response [20], which may partly explain the low infection rate observed.

The difference in specialization between the students from the Faculty of Public Health and the Faculty of Sciences could have influenced their exposure to SARS-CoV-2. Public Health students, due to their training in health centers, were more likely to encounter SARS-CoV-2-positive cases, increasing their infection risk [31]. This elevated risk is further underscored by findings from Lebanon, where a study conducted in 2020 before any vaccines or established treatments for COVID-19 were available revealed a seropositivity rate of 19.7% among 203 healthcare workers [32]. This indicates a significant level of exposure to the virus among healthcare workers in close contact with COVID-19-positive cases, similar to the increased risk faced by the students of the Faculty of Public Health during their training. Nonetheless, the overall SARS-CoV-2 prevalence observed here was very low, and the two positive students were from different faculties.

Finally, the weak surveillance of SARS-CoV-2 in Lebanon also substantially limited genomic characterization of circulating variants. From May to July 2023, 28 sequences from Lebanon were filtered according to the collection date from the GISAID database, with only 4 from June (as of 10 February 2024). Herein, one of the two SARS-CoV-2-positive cases was identified as infection with Omicron BA.2 by qPCR; this case could be BA.2 or XBB subvariant because XBB is a recombinant of two BA.2 sublineages, BA.2.10.1 and BA.2.75 [33]. The predominant Nextclade Pango lineage from May to July 2023 in Lebanon was XBB.1.5* (XBB.1.5 and its descendant XBB.1.5.20), representing 28.5% (8/28) of the sequences, followed by XBB.1.9.1 14.3% (4/28). While one BA.2 sequence was obtained in May 2023, almost BA.2 sequences from Lebanon were collected in 2022. Therefore, the detected case was probably with an XBB.1.5 sublineage (Nextstrain, clade 23 A), an XBB subvariant that has prevailed worldwide since late February 2023 and was characterized by a higher effective reproduction number than other XBB subvariants [34].

Regarding the second student who yielded negative results in the four-qPCR assays specific for Omicron subvariants, this may be due to several factors. First, the variant might not be one of the four tested Omicron subvariants. Second, SARS-CoV-2 variants might not be detected by specific qPCR assays if they have mutations in the targeted genomic regions, affecting primer and probe binding. Additionally, genetic variability and novel recombination of the virus can render existing assays ineffective if they were not designed to detect these changes.

To ensure transparency and provide a comprehensive perspective on our findings, it is essential to acknowledge several limitations of this study. The reliance on self-reported data to determine that 214 individuals had no prior COVID-19 infection may have overlooked previous cases. Furthermore, the lack of precise timing for past infections and the absence of data on serum antibody levels in 208 vaccinated individuals limits our ability to fully understand factors contributing to the observed low infection rate, such as recent immunity or vaccination effectiveness. Meanwhile, a recent study has revealed that ~70% of vaccinated participants with two doses against SARS-CoV-2 in North Lebanon maintained their high immune response (>249 U/mL) between 14 to 30 days after the second dose and 6 months after [20]. The present study also highlights that 41.74% of students in scientific majors remain unvaccinated, and 9.12% of the vaccinated are unaware of their vaccine type. This underscores the critical need for increased awareness about vaccination to curb virus transmission and protect public health. Our study has a cross-sectional design with a short time frame, which could affect our conclusions about the appropriateness of presential over online teaching. The study was also impacted by the official academic recess in August at the Lebanese University, which impeded our progress and resulted in inadequate tracking of participants. Studies investigating longer time frames are, thereby, essential to adequately capture the variation in infection prevalence and the dynamics of COVID-19 transmissions. Finally, our research relies primarily on questionnaires as the primary method of data collection. Despite our considerable efforts in designing and formulating open and closed questions, there remains a potential for inaccuracies or misunderstandings in the respondent’s interpretations, which could impact the validity of the data.

In summary, the present survey, the first of SARS-CoV-2 prevalence among students at a Lebanese university, suggests that students going to healthcare facilities for training or work may be particularly more exposed to the virus and may be asymptomatic carriers due to their younger age and medical status, which could be worth considering in the prevention of future waves of COVID-19 in Lebanon. It also proved a low prevalence in June among asymptomatic university students in Tripoli, Northern Lebanon, and the occurrence of BA.2/XBB Omicron subvariant. This low infection rate may be attributed to low COVID-19 incidence, vaccination-induced immunity, past infections, the younger population, and/or potential high participation rates among asymptomatic students due to the stigma associated with testing positive among symptomatic students. Moreover, as crowded environments contribute to the spread of the virus, adherence to preventive measures such as avoiding contact with infected individuals, wearing masks, maintaining social distance, hand washing, and not sharing objects remain crucial. Regular surveys on COVID-19 and other respiratory infections with longer time frames are essential to manage educational settings during respiratory infection crises.

## Figures and Tables

**Table 1:** Sociodemographic and health-related data of the studied samples.

Characteristic	Total (N = 357)	
	N	%
Sex		
Female	243	68.07
Male	114	31.93
Age-groups		
18–23 years	346	96.92
24–30 years	11	3.08
Marital status		
Single	347	97.2
Married	8	2.24
Divorced	2	0.56
Nationality		
Lebanese	351	98.32
Syrian	5	1.4
Palestinian	1	0.28
District of residence		
Tripoli	164	45.94
Akkar	70	19.61
Menieh-Denieh	56	15.69
Koura	30	8.4
Zgharta	20	5.6
Batroun	8	2.24
Jbeil	4	1.12
Bcharri	3	0.84
Bekaa	1	0.28
Kesrwan	1	0.28
Blood group		
A	172	48.18
B	47	13.17
AB	18	5.04
O	120	33.61
Smoking status		
Never smokers	336	94.12
Ex-smokers	1	0.28
Current smokers	20	5.6
Health condition !		
Yes	24	6.72
No	333	93.28
Current respiratory infections		
Present	31	8.68
Absent	326	91.32
Past SARS-CoV-2-infection status		
Yes	143	40.06
Symptomatic	98	68.53 *
Hospitalization	4	4.08 **
Long term symptoms	26	26.53 **
No	214	59.94
Coronavirus vaccine status		
Yes	208	58.26
Pfizer	183	88 ***
AstraZeneca	4	1.92 ***
Pfizer + AstraZeneca	2	0.96 ***
NA	19	9.12 ***
No	149	41.74

(*): The percentage is calculated based on the total number of students with previous infections (143) as 100%. (**): The percentage is calculated based on the students who showed symptoms as a total. (***): The percentage is calculated based on the vaccinated students as a total. (!): Chronic heart disease (other than blood pressure), blood pressure, diabetes, asthma, chronic pulmonary disease, chronic liver disease, chronic kidney disease, chronic neurological disease, immunosuppression, and cancer. NA: not assigned.

## Data Availability

The data presented in this study are available on request from the corresponding author. The data are not publicly available due to ethical restrictions.

## Supplementary Materials

The following supporting information can be downloaded at https://www.mdpi.com/article/10.3390/pathogens13100872/s1, Table S1: Primers and probes for the following Omicron subvariants.
