# Supplementary material for: A Cross-Sectional Study Revealed a Low Prevalence of SARS-CoV-2 Infection among Asymptomatic University Students in Tripoli, North Lebanon
Source: Pathogens. 2024 Oct 3;13(10):872. doi: 10.3390/pathogens13100872 (PMC11510414; doi:10.3390/pathogens13100872)
Supplement: Supplementary file 1 [file pathogens-13-00872-s001.zip › pathogens-3145964-supplementary.pdf]

APPENDIX

Table S1: Primers and probes for the following Omicron subvariants.

Omicron subvariants	Targeted gene	Primers (in 5'-3' orientation)	Probes (in 5'-3' orientation)
Omicron BA.1	S (spike encoding gene)	(F): CCTTGTAATGGTGTTGAAGGTTTT<br>(R): CTGGTGCATGTAGAAGTTCAAAAG	6VIC-TTTACGATCATATAGTTTCCGACCC
Omicron BA.2	S (Spike encoding gene)	(F): TCTGCTTTACTAATGTCTATGCAGATTC<br>(R): CGCAGCCTGTAAAATCATCTG	6FAM-AGAGGTAATGAAGTCAGCCAAATCGC
Omicron BA.4	ORF7a	(F): TCAAAAGAAAGACAGAATGATTGAAC<br>(R): CTTGCAGTTCAAGTGAGAACCA	6FAM-CTATTTTTGCTTTTTAGCCTTTCTGC
Omicron BA.5	M (Membrane protein encoding gene)	(F): CCTGATCTTCTGGTCTAAACGAA<br>(R): TTCCATTGTTCAAGGAGCTTTT	6FAM-ATGGCAAATTCCAACGGTACTATT

(F): Forward primer, (R): Reverse primer
